# Women’s experience with receiving advice on diet and Self-Monitoring of blood glucose for gestational diabetes mellitus: a qualitative study

**DOI:** 10.1080/02813432.2021.1882077

**Published:** 2021-02-08

**Authors:** Maria Helmersen, Monica Sørensen, Mirjam Lukasse, Hely Katariina Laine, Lisa Garnweidner-Holme

**Affiliations:** a Faculty of Health Sciences, Department of Nursing and Health Promotion, Oslo Metropolitan University, Oslo, Norway; b Faculty of Health and Social Sciences, Centre for Women’s, Family and Child Health, University of South-Eastern Norway, Kongsberg, Norway; c Department of Obstetrics, Oslo University Hospital, Oslo, Norway; d Institute of Clinical Medicine, Faculty of Medicine, University of Oslo, Oslo, Norway

**Keywords:** Gestational diabetes mellitus, qualitative research, primary health care, secondary health care, self-monitoring, care coordination

## Abstract

**Objective:**

We aimed to explore how women with gestational diabetes mellitus (GDM) experience advice about diet and self-monitoring of blood glucose received in primary health care (PHC) and secondary health care (SHC) with a focus on how women perceived the care coordination and collaboration between healthcare professionals.

**Design, setting and subjects:**

Individual interviews were conducted with 12 pregnant women diagnosed with GDM. Six women had immigrant backgrounds, and six were ethnic Norwegian. Women received GDM care in the area of Oslo, Norway. Interviews were analysed using thematic analysis.

**Results:**

Women described feeling shocked when they were diagnosed with GDM and feeling an immediate need for information about the consequences and management of GDM. Most of the women felt that their general practitioner (GP) had too little knowledge about GDM. Women with an immigrant background felt that the PHC midwives provided them with sufficient dietary advice related to GDM. Ethnic Norwegian women appreciated receiving more individually tailored dietary advice in SHC. Self-monitoring of blood glucose influenced women’s daily lives; however, they perceived the training in PHC and SHC as adequate. The women experienced poor collaboration between healthcare professionals in PHC and SHC, which implied that they sometimes had to initiate follow-up steps in their GDM care by themselves.

**Conclusions:**

Ideally, women diagnosed with GDM should meet healthcare professionals with sufficient knowledge about GDM as soon as possible after being diagnosed. The collaboration between healthcare professionals involved in the care of women with GDM should be improved to avoid having women feel that they need to coordinate their own care.

**KEY POINTS**
**Current awareness**
•The management of gestational diabetes mellitus requires appropriate follow-up by healthcare professionals
**Main statements**
•Pregnant women’s need for information about the consequences and management of gestational diabetes mellitus was highest immediately after diagnosis
•Women perceived that they received more individually tailored information about diet and self-monitoring of blood glucose in secondary health care compared to primary health care
•Women felt that general practitioners had insufficient knowledge about gestational diabetes mellitus
•Based on our results, care coordination and collaboration between healthcare professionals involved in the care of women with gestational diabetes mellitus should be improved

## Introduction

Gestational diabetes mellitus (GDM) is defined as carbohydrate intolerance resulting in hyperglycaemia of variable severity with onset or first recognition during pregnancy [1]. The prevalence of GDM is increasing globally and ranges between 1.8%–31.5%, depending on the screening procedure and population characteristics [2]. According to the Norwegian Medical Birth registry, the prevalence of GDM among women giving birth in Norway in 2018 was 5.0% [3]. A cohort study in a district in Oslo identified GDM in 13% of all women, 11% in ethnic Norwegians, and 12%–17% in groups of non-European origin [4]. Risk factors for developing GDM include overweight and obesity, advanced maternal age, a family history of diabetes, GDM in a previous pregnancy and ethnicity [5]. Even though GDM resolves in most women after they give birth, its development may affect the future health of both mothers and children [6,7].

A healthy diet and stable blood glucose levels throughout pregnancy can prevent complications during birth and adverse health outcomes for the mother and the newborn child [8]. First-line management of GDM involves dietary advice based on women’s regularly measured blood glucose levels [9]. About 85% of diagnosed women can manage GDM with lifestyle changes, such as healthy eating and physical activity, without the need for oral antidiabetic therapy or insulin [10]. However, lifestyle changes presuppose knowledge, motivation and follow-up by healthcare professionals [11].

In Norway, new guidelines for the management of GDM were implemented in 2017 which involved a shift in responsibilities from secondary health care (SHC) to primary health care (PHC) [9]. According to the new guidelines, women with mild GDM should primarily be followed up by GPs and/or midwives in PHC [9]. Only women with additional medical risk factors in combination with GDM or women who do not reach the treatment target for blood glucose levels are to be referred to diabetes outpatient clinics in SHC [9]. It is recommended that all women receive written and verbal dietary advice and training in self-monitoring of blood glucose from healthcare professionals in PHC or SHC [9]. Little is known about how women receive this GDM follow-up. Studies among women with GDM in the UK and Australia highlight the challenges in changing lifestyle through standard GDM care, including time constraints and women’s emotional response after diagnosis [12,13]. Women from disadvantaged and immigrant communities are the most at risk of misunderstanding and mismanaging GDM [14,15].

Care coordination and collaboration between healthcare professionals is an important factor in enhancing the care of women with GDM [12,16]. To our knowledge, this is one of the first published studies investigating pregnant women’s experiences of receiving GDM care by different healthcare professionals [12]. Thus, the main aim of this study was to explore how women with GDM experience advice about diet and self-monitoring of blood glucose from both PHC and SHC. We focused on care coordination and on how the collaboration between PHC and SHC is perceived among women with GDM.

## Materials and methods

### Recruitment and participant characteristics

We conducted individual face-to-face interviews with 12 women diagnosed with GDM. Table 1 provides the women’s background information as well as from whom and where they received GDM care. We applied a purposive sampling strategy, aiming to include women who fulfilled the following inclusion criteria: 1) currently pregnant; 2) diagnosed with GDM; 3) experiences with GDM care in PHC and SHC; 4) Norwegian speaking. The women were recruited health professionals at a diabetes outpatient clinic in Oslo, Norway (*n* = 9), and a municipal mother and child health centre (MCHC) in the area of Oslo, Norway (*n* = 2). The health professionals forwarded women’s contact information to MC who contacted them to schedule the interview. One participant contacted MH after advertising for the study in a closed Norwegian Facebook group for women with GDM. The women had either immigrant backgrounds (*n* = 6) or were ethnic Norwegian (*n* = 6). Almost all the women (*n* = 11) were followed up for their GDM in PHC before being referred to diabetes and/or maternity outpatient clinics in SHC. In SHC, women received advice about diet and self-management of blood glucose by nurses specialized in diabetes. Only one of the women was not followed up in SHC. Four of the women were treated with insulin and/or metformin.

**Table 1.** Characteristics and follow-up of the women.

Participant	Age	Education*	Ethnicity	Gestational week when diagnosed with GDM	Previous GDM	Insulin use	Follow-up
1	34	High	Norwegian	23	No	Yes	Midwife and GP (PHC) and SHC
2	32	Middle	Immigrant background	27	Yes	No	Midwife and GP (PHC) and SHC
3	36	Middle	Immigrant background	24–28	No	Yes	Midwife and GP (PHC) and SHC
4	24	Low	Norway	25	No	Yes	GP (PHC) and SHC
5	31	High	Norway	24	No	No	Midwife and GP (PHC) and SHC
6	41	High	Norway	26	Yes	No	Midwife and GP (PHC) and SHC
7	28	Middle	Immigrant background	19	No	No	GP (PHC) and SHC
8	36	High	Immigrant background	31	No	No	Midwife and GP (PHC) and SHC
9	28	High	Immigrant background	26	No	No	Midwife (PHC)
10	28	Middle	Norway	29	No	No	Midwife and GP (PHC) and SHC
11	30	High	Immigrant background	31	No	No	Midwife and GP (PHC) and SHC
12	28	High	Norway	25	No	Yes	Midwife and GP (PHC) and SHC

*Education was categorized as high (3–5 years at university or university college level); middle (8–13 years) or low (1–7 years).

### Data collection

A semi-structured interview guide was developed by the research group for data collection (Supplementary material). One pilot interview was conducted to see how well the interview guide elicited information. As this practice-run led to only minor adjustments (e.g. changes of wording), the pilot interview was included in the final analysis. The main topics in the final interview guide are presented in Appendix 1. The first author (MH) conducted the interviews. MH was a master student in public health nutrition and did not have any experience in qualitative interviewing and only limited knowledge about GDM. Thus, MH was closely followed-up by the last author, an experienced researcher within qualitative interviews among women with GDM (LGH). The interviews lasted from 15 to 45 min and took place in the women’s preferred locations. Four women were interviewed at home, one woman at a local mall, and one on the premises of Oslo Metropolitan University (OsloMet). The remaining six interviews were held at the diabetes outpatient clinic. The interviews were conducted and transcribed between September 2019 and January 2020.

### Data processing and analysis

All interviews were audiotaped, transcribed and analyzed using Braun and Clarkes six-step thematic analysis [17] as follows: 1) transcripts were read and re-read; 2) initial codes were developed by identifying and highlighting meaningful text in the dataset that were relevant to our research question; 3) codes were grouped into meaningful sub-themes and labeled under main themes; 4) the sub-themes and main themes were reviewed; 5) the comprehensiveness of the main themes and whether they worked in relation to the other themes were evaluated; and 6) the results were reported. Analysis was carried by MH and LGH. MS was involved to review the sub-themes and to secure the comprehensiveness of the main themes. MS was responsible for the establishment and implementation of the guidelines for women with GDM at the Norwegian Directorate of Health and had good knowledge about GDM. The other authors critically reviewed the analysis and agreed with the findings. Relevant citations were translated from Norwegian to English.

## Results

Overall, the women stated that they were satisfied with the care they had received in managing their GDM. However, analysis revealed perceived challenges and aspects for improvement presented in Table 2.

**Table 2.** Summary of sub-themes and main themes.

Sub-themes	Main themes
Reactions to being diagnosed with GDM	Shocked to be diagnosed with GDM Difficult to accept the diagnosis Worries for their baby(s) Need for more information about GDM Blamed themselves for GDM
Experience with dietary advice in PHC and SHC	Healthcare professionals as an important source for information about diet Good agreement of dietary advice between different healthcare professionals in both PHC and SHC Felt that the GP (PHC) had little time and knowledge about GDM and diet Immigrant women’s perceptions of dietary advice by the midwife and health care professionals in SHC Acknowledged written dietary information Ethnic Norwegian women often asked for more detailed dietary advice
Experience with training in self-monitoring of blood glucose in PHC and SHC	General satisfaction with training in self-monitoring of blood glucose Felt that they got more detailed information about self-monitoring of blood glucose in SHC More professional follow-up in SHC Institutional variations in how often blood glucose should be measured Surprised to have to measure blood glucose so often Perceived self-monitoring of blood glucose as a simple and logic consequence of the diagnosis Perceived self-monitoring of blood glucose as tiring Self-monitoring of blood glucose affected their daily lives Felt uncomfortable with too high BGL
Experiences of care coordination and collaboration between healthcare professionals in PHC and SHC	Lack of collaboration between PHC and SHC Lack of collaboration within PHC Good inter-professional collaboration in SHC Positive experiences with referral from PHC to SHC Women felt responsible to initiate follow-up after diagnosis

### Reactions to being diagnosed with GDM

Many women were shocked to be diagnosed with GDM. The diagnosis appeared to be more difficult to accept for women who did not consider themselves at risk for developing GDM, compared to those women who were aware of their risk.

The women expressed an immediate need for information about GDM after being diagnosed as they felt that they had no or little knowledge about the consequences and management of GDM. They wanted to know why they developed GDM. Some felt ashamed and blamed themselves for developing GDM, as described by a woman who had not had GDM previously:

When I was first told that I had gestational diabetes, I blamed myself … That it was me who had a bad diet, me who did not exercise enough. (P10)

### Experience with dietary advice

The women often described midwives in PHC and nurses specialized in diabetes in SHC as important sources for dietary advice related to GDM. They also experienced good consistency of dietary advice between healthcare professionals in PHC and SHC. Still, several women used other sources for nutrition-related information, such as the Internet, family and friends. The reason for this was that several women had questions that were not answered during the consultations, as described by a woman who had received care in both PHC and SHC:

I have also looked up some information myself and have joined a Facebook group called *laughs a bit* GDM, ehh so I have in a way done as good as I can, to get into it, with diet and stuff, but I think my GP was probably not the best at informing and follow-up. (P1)

Women felt that their GPs had little knowledge about GDM and about what to eat and that they did not receive the answers they needed. None of the women with an immigrant background experienced difficulties in understanding healthcare professionals’ dietary advice. One of the women reported that her GP had provided her with dietary advice related to GDM in her own language. However, the woman did not perceive that she had received sufficient information:

Avoid sugar. No sweet fruit, which contains a lot of sugar. Don't eat stuff like that… He gave me information, it was not enough for me (P7).

Women with an immigrant background acknowledged the dietary advice from their midwives in PHC. Ethnic Norwegian women appreciated receiving more individually tailored dietary advice in SHC. The women often stated that healthcare professionals should have more time during their consultations to provide them with more individually tailored advice.

Many women received written materials from midwives in PHC. Women experienced this as PHC healthcare professionals not having enough time to explain it directly to the women. Especially, ethnic Norwegian women did not perceive that the written information was sufficiently tailored to their individual needs, e.g. how different foods may affect their blood glucose values.

### Experience with training in self-monitoring of blood glucose

The women were satisfied with how they were trained to self-monitor their blood glucose and did not report difficulties in understanding the training they received from healthcare professionals. However, they often reported that self-monitoring their blood glucose affected their daily lives because they had to plan when and where to measure and had to eat according to the measured values.

In both PHC and SHC, the women received written and verbal information about how often and when to measure their blood glucose. They perceived that they received more detailed information about self-monitoring their blood glucose in SHC compared to PHC; for example, in SHC, they received an explanation about why and how diet can affect their blood glucose levels. They also stated that SHC healthcare professionals seemed more professional than GPs or midwives in PHC and that the information they received from SHC was more individualized.

How often women had to measure their blood glucose varied between 2 and 5 times a day, depending on the individual woman and where and by whom they were followed up. Several women reported that they were told to measure their blood glucose more frequently by healthcare professionals in SHC compared to PHC.

Most of the women were surprised that they had to start measuring their blood glucose. Some women perceived the measurement as a logical consequence of their diagnosis. Others, mostly women with an immigrant background, did not like the idea of having to start self-monitoring their blood glucose and considered it a burden. However, many women found that blood glucose management went smoothly after some time and were motivated to monitor their blood glucose for the sake of their baby, as described by a woman who also had to use insulin:

No, I did. But I had to, I had no choice. You have no choice anymore, you just have to … Eh, if you don't, then it will affect the baby, and everything affects the baby (P4).

The women commonly mentioned that they felt uncomfortable with too high blood glucose values and that, in these situations, they needed more specific dietary advice, especially from the GP and midwife in the PHC, about how blood glucose can be affected by different foods.

### Experience of care coordination and collaboration between healthcare professionals in PHC and SHC

The women in this study often experienced insufficient care coordination and collaboration between healthcare professionals in PHC and SHC. Some women felt that they had to coordinate their own care owing to a lack of communication between health care professionals in PHC and SHC. For instance, a woman complained that her GP did not follow up on a message from SHC that she should start with insulin. Others had to call the GP to ask about the status of her referral to the hospital.

In addition, some women felt that the GP and the midwife in PHC did not collaborate well. This resulted in some women getting information twice and some not receiving any information at all, as an ethnic Norwegian woman commented:

GDM? She (the midwife in PHC), didn’t talk that much about it. She figured I had been at the GP’s office, so maybe she counted on the GP having provided me with information. But I am thinking that it has something to do with the communication between the GP and the midwife (P6).

Compared to PHC, the women perceived a better collaboration between healthcare professionals in SHC, where they often had successive consultations on the same day with a gynecologist, a nurse specializing in diabetes and a midwife.

## Discussion

This study explored the experiences of women with GMD who received dietary advice and training in self-monitoring their blood glucose in Norwegian PHC and SHC. Overall, the women were satisfied with the care they received to help them manage their GDM. Most of the women were shocked when they were diagnosed with GDM and expressed an immediate need for information about the consequences and management of GDM. The women frequently perceived that their GP had little knowledge about GDM. We found differences in the women’s satisfaction with the dietary advice they received depending on their ethnic backgrounds: women with an ethnic Norwegian background asked for more specific and individually tailored advice than women with an immigrant background. All the women felt that this specific need appeared to be better addressed in SHC. According to the women, care coordination and cooperation between healthcare professionals in PHC and SHC should be improved.

Several previous studies have investigated how pregnant women perceive GDM care [13,15,18]. A study of antenatal consultations between midwives and their clients at four diabetes clinics in Norway found that most women, similar to our study, experienced the advice given in SHC about self-monitoring of blood glucose to be adequate [18]. A qualitative study conducted with non-Western immigrants with GDM explored the hospital-based information they were given about GDM and how they integrated this information into their daily lives. Participants with low health literacy and poor Danish language skills struggled to implement the recommended lifestyle changes [15]. Interestingly, the women with an immigrant background in our study did not report any challenges in understanding and following the advice they received about diet and blood glucose monitoring. We found only one study that investigated women’s experiences with follow-up by different healthcare professionals [12]. This qualitative study among women with GDM in south London, UK, emphasizes that GDM care benefits from good collaboration between healthcare professionals [12]. According to the women in our study, collaboration between healthcare professionals in PHC and SHC should be improved.

Women’s experience of feeling shocked by a GDM diagnosis has been described in other studies [19–21]. In our study, the women who were shocked by their diagnosis expressed an immediate need for more knowledge about GDM. Studies have shown that women with GDM often had little knowledge about GDM prior to diagnosis [22–25]. Knowledge about GDM can have an impact on the extent to which a woman follows health and treatment recommendations [26]. Women in our study perceived that their GP’s knowledge about GDM needs to improve compared to midwives’ knowledge, in order to meet their need for information.

The women in our study perceived that self-monitoring their blood glucose interfered with their daily lives. A qualitative study among women with GDM in New Zealand showed that some women disliked the change of focus from pregnancy to their blood glucose levels [21]. The study further showed that women developed barriers to manage their blood glucose levels owing to inconsistent advice from healthcare professionals and lack of information in the women’s first language [21]. Time pressure during consultations and limited comprehension of training requirements may be barriers to effectively teaching women to self-monitor their blood glucose [13], but these barriers may be overcome by improving communication between women and healthcare professionals [13]. In our study, performing self-monitoring of their blood glucose affected the women’s daily lives because they had to plan more carefully than before when and what to eat. However, they were satisfied with the training they received.

Other studies indicate that immigrant women can have problems with understanding and following dietary advice and training in self-monitoring their blood glucose [14,15,20]. Even though we did not find differences in how the women experienced training in self-monitoring their blood glucose and dietary advice depending on their ethnic background, the Norwegian women more often asked for more specific and individually tailored dietary advice than the women with other ethnic backgrounds. This might be due to their higher educational level compared to the women with an immigrant background in this study. Healthcare professionals could provide women with individual meal plans that take individual food preferences and blood glucose values into account [27]. However, as outlined by the women in this and another study, the limited time for consultations might be a barrier to providing individually tailored advice [27].

### Strengths and limitations

The findings from this study might be especially valuable for policymakers and healthcare professionals in countries where responsibility for the care of women with GDM is shifting from SHC to PHC. We recruited women of various ethnic backgrounds, and some of the women recruited had limited Norwegian language skills, which may have affected the interviews. The interviewer often repeated what the women said to ensure mutual understanding. Many of the women in this study were recruited by and followed up by the same healthcare professionals in SHC; thus, our findings may be context-specific and cannot be generalized. Typically for qualitative studies, the educational background and personal experiences might affect the data collection and interpretation of the results. However, we have involved researchers with varying experience with qualitative studies and GDM to limit the possible bias of a single researcher’s preconceptions on the data collection and interpretation of the results. Neither of the authors work with GDM patients and had no preconceived ideas about how women perceive their care. We aimed to include women with experience of being followed-up in both PHC and SHC. We did not ask participants about the precise number of received consultations, however, all of the women appeared to have experiences from several consultations. Even though one of the women received only PHC care, her interview was included as this woman provided important insights about inter-professional collaboration within that care.

## Conclusions and implications for policy and practice

This study showed that women with GDM were generally satisfied with the GDM care they received, although some women asked for more individually tailored dietary advice. Women diagnosed with GDM should meet with healthcare professionals who have sufficient knowledge about GDM as soon as possible after being diagnosed. The collaboration between healthcare professionals involved in the care of women with GDM should be improved to avoid having the women themselves feel the need to coordinate their own care.

## Supplementary Material

Supplemental Material
