# Supplementary material for: Women’s experience with receiving advice on diet and Self-Monitoring of blood glucose for gestational diabetes mellitus: a qualitative study
Source: Scand J Prim Health Care. 2021 Feb 8;39(1):44–50. doi: 10.1080/02813432.2021.1882077 (PMC7971282; doi:10.1080/02813432.2021.1882077)
Supplement: Supplemental Material [file IPRI_A_1882077_SM0511.docx]

**Interview guide:**

Introduction question:

Have you heard about gestational diabetes before you were diagnosed?

Can you tell me something about your general experiences with follow up of your gestational diabetes (have you been followed up by your GP/midwife or/and the outpatient clinic at the hospital?)

**Main topics and probing questions:**

- Experiences of getting information about a healthy diet

What do you think about the dietary advice you have received during your GDM care?

Who/what was your most important source for advice about a healthy diet?

How did you experience to get information about diet from different health care professionals?

Did you experience any difficulties in getting information about diet from different health care professionals?

Was there something you missed when receiving information about diet?

- Experiences in getting training in self-monitoring of blood glucose

What did you think when you got to know that you had to measure your own blood glucose?

Who trained you in self-monitoring of blood glucose? How did you experience the training?

How do you feel the self-monitoring of blood glucose is working?

Can you tell me something about your experiences in getting training from different health care professionals?

- Women’s informational needs about diet and self-management of blood glucose

In what way do you think health care professionals could in best way adapt information about diet and self-monitoring blood glucose to you as a pregnant with gestational diabetes?

- Experiences of care-coordination and collaboration of different health care professionals involved in GDM care

How did you experience the collaboration between different health care professionals in providing you with information about diet and training in self-monitoring of blood glucose?

Closing question:

What was your motivation participating in this study?
